# Developing wipe sampling strategy guidance for assessing environmental contamination of antineoplastic drugs

**DOI:** 10.1177/10781552221118535

**Published:** 2022-08-04

**Authors:** Susan Arnold, Matthew Jeronimo, George Astrakianakis, Miranda Kunz, Ashley Petersen, Carole Chambers, Darcy Malard Johnson, Emily Zimdars, Hugh W Davies

**Affiliations:** 1 43353 School of Public Health, University of Minnesota, Minneapolis, MN, USA; 2 120479 School of Population and Public Health, University of British Columbia, Vancouver, British Columbia, Canada; 3 Alberta Health Services, Calgary, Alberta, Canada; 4 M Health Fairview, Minneapolis, MN, USA

**Keywords:** exposure assessment, wipe sampling, antineoplastic drug, surveillance

## Abstract

Surveillance for environmental contamination of antineoplastic drugs has been recommended by authoritative bodies such as the United States Pharmacopeia and the National Association of Pharmacy Regulatory Authorities. Clear guidance is needed on how to develop sampling strategies that align with surveillance objectives efficiently and effectively. We conducted a series of simulations using previously collected surveillance data from nine cancer treatment centers to evaluate different sampling strategies. We evaluated the impact of sampling 2, 5, 10, or 20 surfaces, at monthly, quarterly, semi-annual, and annual frequencies, while employing either a random or sentinel surface selection strategy to assess contamination by a single antineoplastic drug (AD) or by a panel of three ADs. We applied two different benchmarks: a binary benchmark of above or below the limit of detection and AD-specific hygienic guidance values, based on 90th percentile values as quantitative benchmarks. The use of sentinel surfaces to evaluate a three-drug panel relative to 90th percentile hygienic guidance values (HGVs) resulted in the most efficient and effective surveillance strategy.

## Introduction

The overarching goal of environmental surveillance, where a clear occupational exposure-health outcome relationship has been identified, is to reduce work-related exposures. These exposure reductions will, in time, reduce the burden of disease associated with the exposure.^1^ Environmental surveillance of hazardous drugs has been recommended by authoritative bodies such as the United States Pharmacopeia (USP) Chapter <800> “*Hazardous Drugs—Handling in Healthcare Settings”,*^2^ NAPRA^3^ and the American Society of Health-System Pharmacists (ASHP)^4^ toward promoting patient and worker safety, and toward environmental protection. Achieving this goal of reducing the health risks associated with occupational exposure to hazardous drugs requires efficient and effective sampling strategies that are informed by quantitative exposure data. Aligning sampling strategies with the sampling objectives promotes more efficient and effective surveillance. Efficient strategies produce the most useful information with the minimum number of samples, while effective strategies ensure that the sampling results accurately address the questions that were asked (e.g. about the exposure potential and relatedly, exposure risks).

Several barriers have limited the development of statistically valid, efficient, and effective occupational hygiene strategies in the surveillance of antineoplastic drugs (ADs). Despite a growing body of literature around environmental surface wipe data, few of these have been large surveillance studies and knowledge gaps persist One significant gap is guidance on where, when, and what to sample and how many samples should be collected. For airborne and physical hazards, statistically based guidance linking sample size with the probability of finding exceedances above occupational exposure limits has been available for a long time^5,6^ but is lacking for surface wipe sampling. This may be due to the lack of standardization across methods that limit the comparison of data and the relatively short history of this kind of surveillance relative to other surveillance methods. Surveillance of inhalation exposures to dust in the mining industry, for example, has been in place for nearly a century. Whereas surveillance of hazardous drugs using surface wipe sampling was only first reported in the literature about 30 years ago.^7,8^ The complex exposure-to-disease pathway, from surface contamination of ADs to dermal contact, uptake, absorption, and distribution, to disease, makes it more difficult to interpret exposure concentration results in a health risk context. The long lag time between exposure to ADs and the most serious, chronic outcomes, adverse reproductive health or occupational cancer, further complicates contextualizing results and may serve as a barrier to wider adoption of this surveillance method. These complexities are barriers to setting occupational exposure limits (OELs). The lack of regulatory or consensus health-based OELs for individual ADs and especially surface-based OELs impedes within- and between-workplace hygiene comparisons,^9^ which may reduce the motivation for monitoring and further contribute to the lack of data. The lack of data to support guidance on how many drugs should be assessed is cited by USP <800> as the barrier to making any recommendations regarding sample size.^2^ Similarly, while Hon et al.^10^ noted that the selection of a single indicator marker when conducting wipe sampling could underestimate the true level of contamination, specific guidance on how many ADs to include when wipe sampling, and which ADs to choose, is lacking. Connor et al.,^11,12^ found that sampling for multiple drugs increases the probability of detection of contamination.

Criteria for the standardization of methods, such as sample collection and analysis and reporting of results, are another important factor.^13^ Sample collection and analysis methods differ between researchers.^14–16^ While there are numerous published surveillance studies, the inconsistent and often insufficient documentation of contextual details related to the work environment and work practices at the time wipe sampling was conducted make it difficult to interpret findings. These differences also make it difficult to pool results across studies that would otherwise allow for a more in-depth statistical analysis.

Without defined departure points from exposure-response studies based on dermal exposures (e.g. no-observable-adverse-effect levels [NOAELs]), the setting of dermal OELs is challenging. Surface wipe limits that are typically derived from these health-based OELs do not yet exist; this would require conversion factors to account for transfer rates from surface to skin, dermal uptake, metabolism, and distribution to target organs.^17^ The lack of health-based OELs also make it is difficult to define the necessary analytical method sensitivity to ensure the limits of detection (LOD) and quantification are adequate for managing health risks associated with these drugs. Limits of detection vary widely depending on the analytical method employed, model and age of analytical instrument, properties of the specific AD, and other factors. This variability can lead to differences in the level of residual surface contamination reported as <LOD.

Hygienic guidance values (HGVs) have been proposed as alternative metrics for evaluating work practices and the work environment relative to potential exposure risks.^18–20^ HGVs, also known as Threshold Guidance Values,^17^ are non-health-based guidelines developed from comprehensive baseline surveys for the assessment of preparatory hygiene practices and safety measures. These guidelines could be used to provide feedback to personnel toward improving their work practices, toward continuous reduction of environmental contamination and worker exposure.^17^

Amidst these knowledge gaps and barriers, USP Chapter <800>: Hazardous Drugs – Handling in Healthcare Settings^2^ outlines practice and quality standards for handling hazardous drugs. Entities handling hazardous drugs are required to incorporate this chapter’s guidance into their health and safety management system.^2^ Among the environmental quality and control standards is the requirement to conduct environmental wipe sampling for hazardous drug surface residues, including ADs: initially, to establish baseline levels and then at least semi-annually, to ‘verify containment’. While Chapter <800> lists several specific surfaces as candidate sampling locations, the list is not comprehensive. It does not provide guidance on which ADs or combinations of ADs should be sampled. There is no instruction on how to identify candidate marker ADs. USP <800> stipulates wipe sampling ‘routinely’ and lists several candidate surfaces (Table 1). It recommends sampling semi-annually or ‘more often as needed’. Citing a lack of studies demonstrating the effectiveness of a specific number or size of wipe samples, the chapter does not offer any specific guidance on sample size or wipe surface area.^2^

**Table 1.** USP chapter <800> recommended surfaces to be wipe sampled.

Location	Surface
*Compounding Pharmacy*	Interior of the BSC and equipment contained in it
	Pass-through chambers
	Surfaces in staging or work areas near the BSC
	Areas adjacent to BSCs (e.g. floors directly under BSC, staging, dispensing area)
*Pharmacy*	Areas immediately outside the HD buffer room or the C-SCA
*Patient administration*	Patient administration areas

BSC: Biological Safety Cabinet also referred to as; C-PEC: containment primary engineering control; C-SCA: containment segregated compounding area.

Sampling strategy guidance derived from quantitative modeling would promote efficient and effective surveillance. The objectives of the work described in this paper are to (i) assess the efficiency and effectiveness of several candidate sampling strategies including USP Chapter <800> and (ii) recommend sampling strategy guidance based on modeled scenarios using real exposure data.

## Methods

Data from a comprehensive surface-wipe surveillance study of nine centers located in Canada and the U.S and conducted over a 12-month period investigating 11 ADs^21^ were used to model scenarios from which sampling strategy effectiveness and efficiency were assessed. Sampling strategy recommendations were developed from these assessments and from previously identified determinants.^15,21^ Strategies modeled included changing the number of surfaces sampled (Table 2) and the frequency with which sampling was conducted. We modeled scenarios where surfaces were randomly selected versus selected based on a sentinel surface strategy (Table 3), which we defined as surfaces most likely to be contaminated with AD residues based on the frequency of detection in our own surveillance data,^21^ previous in-house monitoring, previous monitoring or reported staff concerns, or surfaces suspected to be contaminated, for example, from peer-reviewed literature. Finally, we modeled all scenarios using the (i) proportion above the LOD and (ii) HGVs as benchmarks.

**Table 2.** Wipe sampling strategies using randomly selected surfaces.

Strategy	Surface location
All 20 surfaces	Pharmacy: n = 10 surfaces Patient Admin: n = 10 surfaces
10 randomly selected surfaces	Pharmacy: n = 5 surfaces Patient Admin: n = 5 surfaces
5 randomly selected surfaces:	Pharmacy: n = 3 surfaces Patient Admin: n = 2 surfaces
2 randomly selected surfaces	Pharmacy: n = 1 surface Patient Admin: n = 1 surface

**Table 3.** Wipe sampling strategies using sentinel surfaces.

Strategy	Sentinel surfaces	Location^a^
All 20 surfaces	All 20 surfaces	
10 sentinel surfaces	BSC work surface HD fridge handle Room temp storage Clean room cart Pass-through tray	Pharmacy
	Storage shelf/tray Side table IV key pump pad Patient door handle Patient toilet handle	Patient Administration
5 sentinel surfaces^b^	BSC work surface HD fridge handle Room temp storage	Pharmacy
	IV key pump pad Patient door handle	Patient Administration
2 sentinel surfaces	HD fridge handle	Pharmacy
	IV key pump pad	Patient Administration

^a^ Surface sample locations were selected from data reported in Jeronimo et al., (2021).

^b^ The five sentinel surfaces strategy aligns with the USP Chapter <800> wipe sampling guidance.

To assess the impact of the number of drugs assessed, the number of surfaces wiped, and the sampling frequency on the likelihood of identifying AD contamination, we simulated sampling using the observed proportions of ADs above the LOD on randomly selected surfaces for the three specific ADs that were detected most frequently in our surveillance study: cyclophosphamide (CP) (16% > LOD), gemcitabine (GEM) (24% > LOD), and paclitaxel (PCX) (13% > LOD).^21^ The surface-specific proportions above the LOD used are given in Table 3 of Jeronimo et al.^21^ Each sampling strategy was replicated to simulate 10,000 sampling events, and the proportion of events for which any drug or individual drugs (CP, GEM, or PCX) were detected was calculated for each combination of sampling frequency and number of surfaces sampled. For example, simulation of a sampling event on an IV pump keypad was based on the proportion above the LOD for the specific drug from all IV pump key pads sampled in Jeronimo et al.^22^ These simulated events allowed for different randomly selected surfaces to be sampled, but it was assumed that, if an event included sampling multiple times per year, the same randomly selected surfaces were sampled each time. Four sampling strategies were evaluated (Table 2).

To evaluate the impact of using ‘sentinel surfaces’, we simulated sampling using observed proportions of drug above the limit of detection (LOD) on different surfaces for three specific drugs: CP, GEM, and PCX, as described above. Sentinel surfaces were selected in this work based on the surfaces with the highest average proportions > LOD for the three ADs of interest (Table 3). For comparison, we included a strategy using all 20 surfaces that were wipe sampled at each location in our surveillance study.

We also examined sampling strategies using alternative metrics defined as HGVs, by substituting the reference 90th percentile concentrations for the LOD. If we define the decision metric as the 90th percentile calculated from the baseline sampling results for each AD, the proportion of surfaces with contamination above this level could be used to evaluate changes in environmental surface contamination over time. We simulated sampling 20, 10, 5, and 2 surfaces for three drugs over 10,000 events with different proportions of ADs above the reference 90th percentile of surface contamination to evaluate this approach. Specifically, we looked at scenarios where 30%, 20%, 10%, or 5% of the surfaces had contamination above the reference 90th percentile for each of the three drugs, simulating the transition from a poorly controlled to a controlled environment one would expect following a continuous improvement program. Since we are modeling the percent above the reference 90th percentile (not the actual 90th percentile for each AD) and the percent above the reference 90th percentile is assumed to be the same for each AD, the results will be the same for each of the three ADs. All simulations were completed in R version 3.6.2.^23^

## Results

We evaluated two approaches to AD surveillance, using the LOD as a binary criterion for exposure (<LOD representative of not exposed, >LOD representative of exposed) and using the reference 90th percentile concentrations as a binary criterion. We tested four sampling frequencies and assessed several strategies using randomly selected surfaces versus sentinel surfaces. For CP, GEM, and PCX, 90th percentile values from pharmacy surfaces were 0.031 ng/cm^2^, 0.007 ng/cm^2^, and 0.003 ng/cm^2^, respectively. In the patient administration areas, the 90th percentile values for CP and GEM were 0.018 ng/cm^2^ and 0.005 ng/cm^2^, respectively. We were not able to establish a 90th-percentile value for PCX in the patient administration areas from our empirical surveillance data due to <10% of sampled surface contaminations being >LOD.^21^

**Figure 1.**
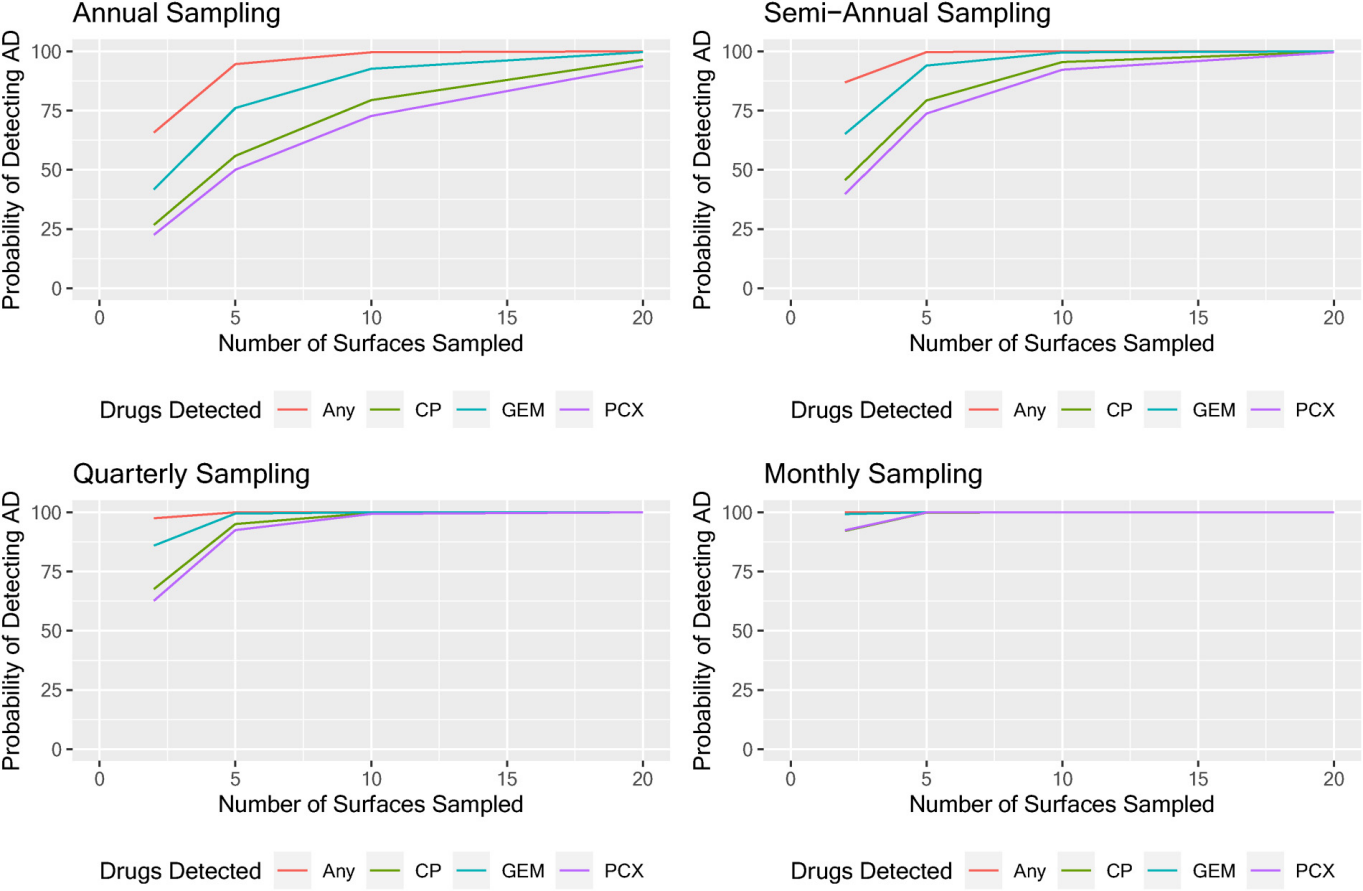
Evaluating the effectiveness of different sampling strategies for wipe sampling antineoplastic drugs (ADs) in clinical settings by sampling 2, 5, 10, or 20 randomly selected surfaces and assessing individual ADs or any AD > LOD annually, semi-annually, quarterly, or monthly.

When surfaces were randomly selected, the likelihood of detecting AD residues >LOD was the highest when surveillance included a panel of three ADs, and when finding any one of the three ADs > LOD was considered to be a positive result. Increasing the number of surfaces tested from two to five, and up to 10 surfaces also increased the likelihood of detecting AD residues. Similarly, the likelihood of detecting AD residues increased with increasing sampling frequency over a year (Figure 1). Although sampling more (either via a larger number of surfaces or more frequently) increases the likelihood of detecting AD residues, there are diminishing returns. For example, when sampling annually with a three-AD panel, there is a large increase in the likelihood of detection when going from two to five surfaces sampled, but a much more modest gain when going from five to ten surfaces sampled. Sampling for only one drug lowered the likelihood, especially when sampling fewer than five surfaces and sampling only annually or every six months, as recommended in USP Chapter <800>.

**Figure 2.**
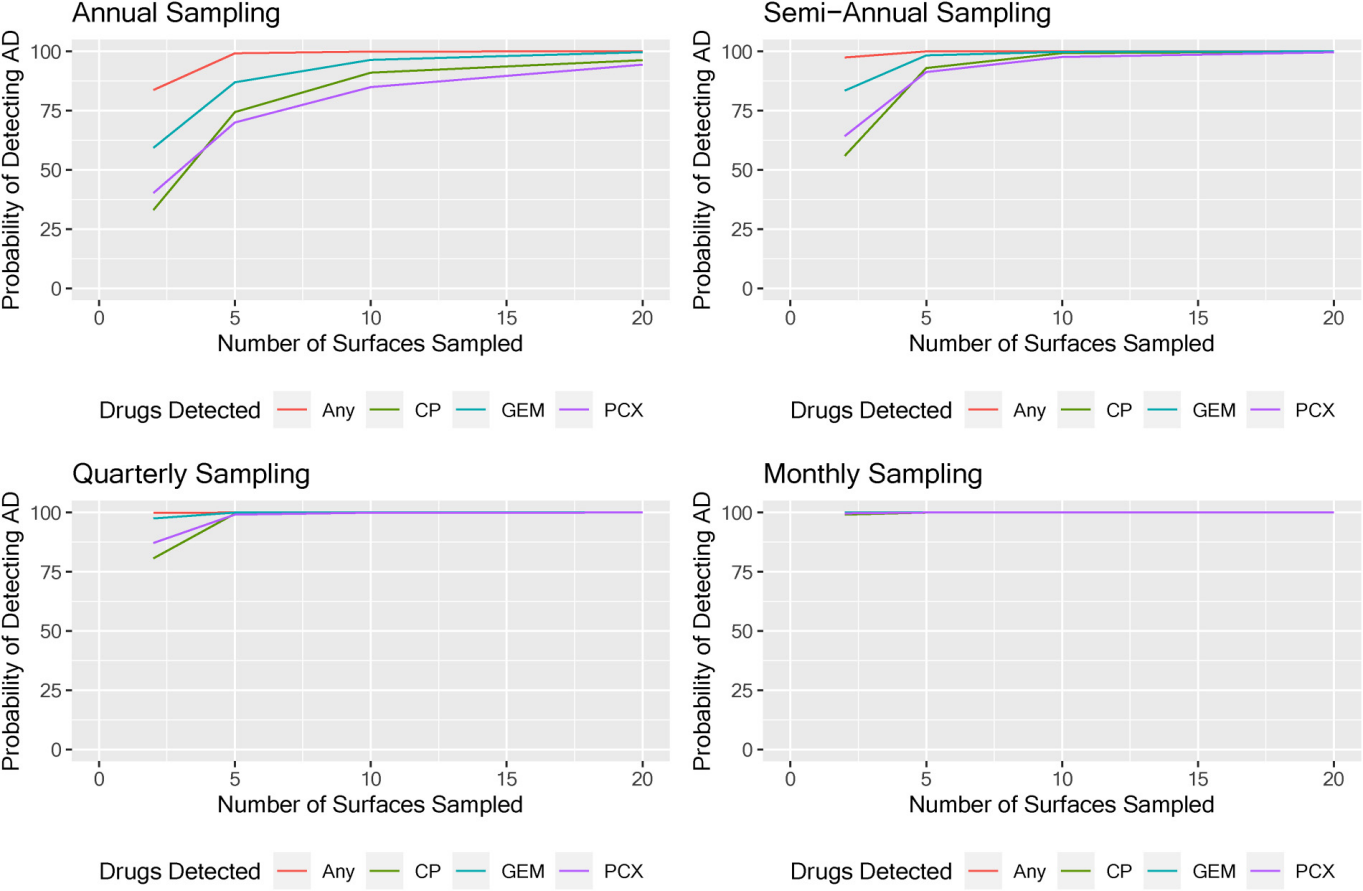
Evaluating the effectiveness of different sampling strategies for wipe sampling antineoplastic drugs (ADs) in clinical settings by sampling 2, 5, 10, or 20 sentinel surfaces and assessing individual AD or any AD > LOD annually, semi-annually, quarterly, and monthly.

Using sentinel surfaces improved the likelihood of detecting surfaces with AD residues > LOD across all four sampling frequencies (Figure 2). Collecting five wipe samples from sentinel surfaces, on a semi-annual frequency as recommended by USP Chapter <800>, and assessing a three-AD panel, resulted in a >99% probability of detecting surface residues present at concentrations >LOD.

**Figure 3.**
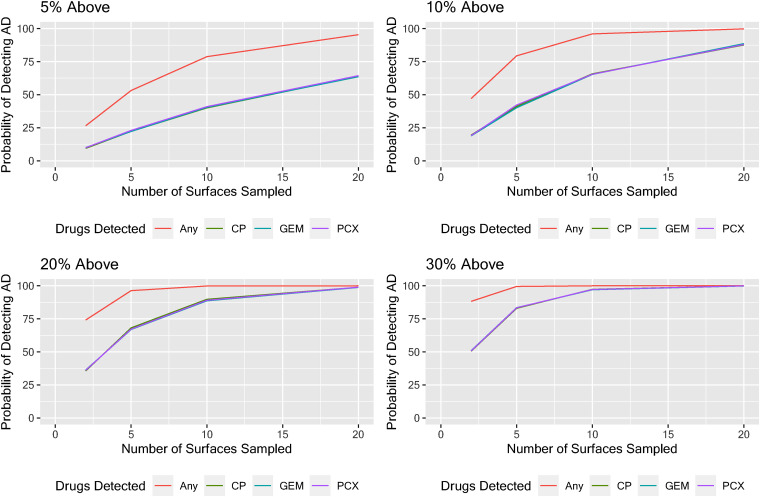
Evaluating the effectiveness of different sampling strategies for wipe sampling antineoplastic drugs (ADs) in clinical settings by sampling 2, 5, 10, or 20 sentinel surfaces and assessing individual ADs or any AD above the reference 90th percentile when the true proportion above the reference 90th percentile is 5, 10, 20, or 30%.

For strategies employing a reference 90th percentile value as the HGV, the greatest likelihood of detecting surfaces with contamination at or greater than the HGV was achieved by analyzing the three-AD panel. Under a continuous improvement scheme, a reduction in surface contamination levels would result in a smaller proportion of samples exceeding the HGV. In our simulation, when the ‘true’ proportion of samples at or above the HGV decreased from 30% to 5% (represented by a lower proportion of surfaces above the HGV), the number of wipe samples needed to achieve a likelihood of detection of at least 90% increased from 5 to 15 (Figure 3).

Based on these results, the USP Chapter <800> recommendations should be augmented to provide guidance on the candidate surfaces to be wipe sampled and recommend a sampling frequency of at least every six months (see Supplemental Materials for Recommended Practice Document for general guidance).

### Wipe sampling guidance for evaluating the effectiveness of controls

In conjunction with a comprehensive hazardous drug safe handling program, augment the approach outlined in USP Chapter <800> by:

- Develop a site-specific guidebook to capture surfaces, a specific location on each surface, and where samples were collected, for future reference. Consider including a site map to show where the sample is located in the work area. Include a digital photo of the sampling location with the wipe sample template in place, to show exactly where the sample will be/was collected.
- Use a sentinel surfaces strategy. Select surfaces from both the compounding pharmacy and patient administration areas for wipe sampling. Use site-specific data, if available, to prioritize surfaces that are frequently touched by a small number of people (e.g. a refrigerator door that a pharmacy tech touches repeatedly) and high-touch surfaces (e.g. a doorknob touched by a multitude of people including staff and patients). These surfaces tend to be associated with a greater likelihood of surface contamination.^16^ Surfaces identified in the literature or other guidance documents, e.g., USP Chapter <800>, are also useful for identifying sentinel surfaces. Since contamination is widely recognized to occur beyond the pharmacy, some ‘high risk’ or sentinel surfaces in non-pharmacy surfaces should be included.
- Collect at least five wipe samples from surfaces known or suspected to have higher levels of ADs. Consider increasing the number of samples to n = 10, to verify effective control in environments known or believed to be well-controlled (<10% above HGV).
- Supplement semi-annual sampling recommended under USP Chapter <800> with *ad hoc* sampling, e.g., post-spill to verify decontamination. Semi-annual sampling increased the likelihood of detecting surface contamination for a three-AD panel, especially when used in combination with a sentinel surface sampling strategy in our study.
- Sample for three or more ADs per wipe sample. Select ADs to be sampled based on the following criteria:
  - Cancer treatments given at the organization’s sites and the ADs associated with those treatments. General guidance on recommended ADs for targeted cancer treatment is available from published sources, including the gray literature.^24^
  - ADs compounded in the 48 h preceding sampling. In our surveillance study,^21^ the amount of drug compounded within the past 48 h was significantly associated with >LOD and contamination (> reference 90th percentile) for the three most frequently detected drugs.
  - Persistence on surfaces: Select ADs that are not light sensitive and are more environmentally stable.^15^
  - Analytical sensitivity: Select ADs with low LOD, that is, low ng to pg level to achieve the best chance of detecting surface ADs, in accordance with ALARA principle.^25^
- Select quantitative benchmarks such as HGVs, using a reference upper tail metric (e.g. reference 90th percentile value). Based on 90th percentile values of surface contamination in the pharmacies evaluated in our study, the HGVs for CP, GEM, and PCX would be 0.031, 0.007, and 0.003 ng/cm^2^, respectively. Track the proportion of ADs exceeding this value over time (e.g. annually) to ensure continuous reduction in surface contamination.

## Discussion

Sampling strategies necessarily differ according to the surveillance objectives. Routine surveillance to confirm adequate control of ADs in accordance with the ALARA principle should follow a strategy that efficiently and effectively detects surface contamination. We conducted surveillance simulations to provide guidance for routine surveillance of AD contamination. Our modeling was informed by a large surface wipe dataset that was collected monthly for 11 drugs over a one-year period from pharmacy and patient administration surfaces in nine centers located in Alberta, Canada, and Minnesota, USA.^21^

The historical use of a single AD as a marker drug, such as CP, may not be the best indicator for every site despite being the drug that is most frequently cited in the literature, especially if it is the singular AD that is surveilled. Our own study suggests that selecting a panel of ADs may be more effective. A panel of at least three ADs, where detecting any one of them was considered a positive result, was a more efficient approach, improving the likelihood of detecting surface contamination compared with any single AD.

Although our evaluation of sampling strategies included using >LOD as a decision metric, caution is advised using this approach since LODs can differ for each AD. In some cases, LODs differ by many orders of magnitude, for example, the reported LOD for CP ranges from 1 pg/cm^2^ (0.001 ng/cm^2^)^26^ to 17.8 ng/cm^2^.^27^ One alternative approach would be to establish HGVs as benchmarks to guide decision-making and follow up. Generic HGVs can simplify the evaluation process by establishing a single value that would be applied to all ADs. A substance-independent HGV of 0.1 ng/cm^2^ has been proposed as an initial target, with stricter AD-specific guidelines being substituted as the surveillance program matures that align with the exposure-risk relationships of these drugs, especially those identified as occupational carcinogens.^19^ This level is much lower than the upper bound contaminant level of 1 ng/cm^2^ indicated in USP Chapter <800>.^2^ However, this may not be the most efficient approach, since ADs have different toxicological thresholds and LOD. Universally applying a generic HGV may misdirect resources to remove less potent ADs instead of focusing on surface contamination of more bioactive ADs.

AD-specific HGVs provide targeted, objective benchmarks for assessing the adequacy of work practices, occupational hygiene, and continuous improvement in reducing potential exposure to ADs. One study^17^ proposed a tiered, AD-specific approach from surface wipe data of platinum (as a marker for the cytostatic drugs cis-, carbo-, and oxaliplatin) and 5-Fluoruracil. In this scheme, AD levels below TGV-1 work practices represented good working practices, whereas levels at or above TGV-2 indicated the need for improving handling practices among pharmacy workers. TGV-1 and TGV-2 values represent the median and 75th percentile contaminant levels for the specific AD. Kiffmeyer et al.^19^ proposed an AD-independent HGV, based on 90th percentile contamination values of eight different ADs for monitoring environmental contamination in pharmacies. HGVs have also been used in the assessment of patient administration areas to protect nursing staff, with HGVs based on 75th percentile values^17^ and 90th percentile AD contaminant levels.^13,18,20^ These 90th percentile value HGVs, ranging from 0.0028 ng/cm^2^ to 0.035 ng/cm^2^, are similar to the 90th percentile HGV value of 0.031 ng/cm^2^ proposed for CP in this work. The 90th percentile level corresponding to the HGV for GEM proposed in this work is consistent with the 90th percentile level of contamination of 0.007 ng/cm^2^ reported in Kiffmeyer et al.^19^ and nearly the same as the 90th percentile value of GEM reported by Chauchat et al.^28^ of 0.006 ng/cm^2^. These proposed HGVs are much lower than those described in Sottani et al.^18^ (3.6 and 0.9 ng/cm^2^ for CP and GEM, respectively).

Establishing HGVs based on upper percentile values requires a relatively large number of samples to account for the inherent variability in contamination levels that is likely to exceed what is practically feasible for many organizations. Publicly available values of surface ADs could serve as reasonable surrogates. This would be most appropriate where the sites included in the published study are similar to the site seeking to use an HGV. The similarities in magnitudes of HGVs for CP proposed here, based on reported 90th percentile values from studies conducted in five countries (Canada, U.S., Italy, Sweden, and Germany) involving 248 sites^13,17,18,21,28^ suggests that these values are generalizable and could serve as initial HGVs for clinical environments. Over time, however, with continuous improvement programs resulting in a reduction of environmental contamination of ADs, and reductions in the proportion of samples exceeding the reference 90th percentile value, periodic adjustment of the HGV would be necessary to reflect these changes.

For many ADs with known or suspected carcinogenic effects, exposures must be controlled to be as low as reasonably achievable until there are health-based occupational exposure limits available. ADs are a suite of disparate chemicals encompassing a wide range of chemical properties and toxicities, making a single OEL to regulate, monitor, and assess all ADs as a single substance impractical. Substance-specific, health-based OELs would address differences in toxicities and potencies and provide the necessary benchmarks against which analytical methods could be optimized to ensure sufficient analytical sensitivity. Further, setting substance-specific OELs would be an important step forward toward addressing the multitude of hazardous drugs, and therefore the potential for exposure to a wide range of ADs, or potential for concurrent exposures. The approach followed by the ACGIH Chemical Substances Threshold Limit Value committee should also be explored, to set OELs (SL-TLVs) for each AD from lowest departure point for a non-cancer outcome; and address cancer outcomes separately by denoting the cancer outcome categorically.

Site mapping is recommended to facilitate network analysis, which could provide a means for studying the propagation of contamination within the unit and elsewhere within the facility. Benchmarking against other sites is also a useful approach for ensuring a site is performing to this standard and potentially identifying common propagation pathways. This is difficult to do right now, due to the lack of a central data repository, range of sampling and analytical methods, and lack of standardization on how results and supporting details are recorded. To address these gaps, we recommend that health regions/regulators create a database to capture surveillance results, along with contextual information to support ongoing research and benchmarking. This database would enable the evaluation needed to standardize on methods and establish wipe surface limits. With defined wipe surface limits, minimum levels of detection for analytical methods can be established.

These recommendations are based on simulations that were informed primarily by one study.^21^ The simulations necessarily involve some simplifying assumptions; for example, we assumed that once a candidate surface was selected that same surface was wipe sampled in all subsequent rounds of sampling. Our simulations were constrained to examining a three-AD panel of CP, GEM, and PCX by the underlying surveillance data set. Nevertheless, the similarity of the surveillance results compared with other published studies suggests that the assumptions are reasonably representative of a range of sites and clinical environments.

## Conclusion

The outcomes from the simulations provide a scientific basis for recommendations that can inform the development of efficient and effective sampling strategies for routine surveillance. Following a sentinel surface approach to evaluate a panel of at least three ADs against AD-specific HGVs resulted in the most effective strategy. Using HGVs to evaluate the adequacy of controls and work practices as part of a continuous improvement focus will help ensure exposures are kept as low as is reasonably achievable.

## Supplemental Material

sj-docx-1-opp-10.1177_10781552221118535 - Supplemental material for Developing wipe sampling strategy guidance for assessing environmental contamination of antineoplastic drugs Click here for additional data file. Supplemental material, sj-docx-1-opp-10.1177_10781552221118535 for Developing wipe sampling strategy guidance for assessing environmental contamination of antineoplastic drugs by Susan Arnold, Matthew Jeronimo, George Astrakianakis, Miranda Kunz, Ashley Petersen, Carole Chambers, Darcy Malard Johnson, Emily Zimdars and Hugh W Davies in Journal of Oncology Pharmacy Practice
