# Supplementary material for: Developing wipe sampling strategy guidance for assessing environmental contamination of antineoplastic drugs
Source: J Oncol Pharm Pract. 2022 Aug 4;29(8):1816–24. doi: 10.1177/10781552221118535 (PMC10687812; doi:10.1177/10781552221118535)
Supplement: sj-docx-1-opp-10.1177_10781552221118535 - Supplemental material for Developing wipe sampling strategy guidance for assessing environmental contamination of antineoplastic drugs [file sj-docx-1-opp-10.1177_10781552221118535.docx]

## Supplemental Materials

**Wipe Sampling – General Guidance**

- Develop a site-specific guidebook to capture surfaces and specific locations on each surface where samples were collected, for future reference. Consider including a site map to show where the sample is located in the work area. Include a digital photo of the sampling location with the wipe sample template in place, to show exactly where sample will be/was collected.
- Document contextual information contemporaneously, including determinant data such as:
  - Drugs compounded on day of and day prior to wipe sampling (which drugs and quantity) to help evaluate the potential for accumulation of the more stable ADs through multiple contaminations
  - Location of surface
  - Surface type (e.g., stainless steel, glass, laminate)
  - Surface area size
  - Surface wipe area
  - PPE compliance (based on the site’s policies and required practices)
- Select a validated wipe sampling method, with known recovery efficiency from the surface types you will be sampling. Align the surface wipe area to meet the sampling strategy objectives, e.g., if the objective is to detect the presence of ADs, it may be desirable to wipe a larger area for larger, flat work surfaces. If the purpose is to evaluate surface residue variability, it may be preferable to collect several wipes with each one covering a smaller surface area at various locations of the same surface. It is important to note that this latter approach will likely be more expensive, since the analysis is usually based on a per sample fee.
- Allow time for staff to get adequate training and become comfortable with wipe sampling techniques, special handling requirements (e.g., refrigerated shipment of samples, shelf life), and shipping procedures.
- Ensure that the laboratory used to analyze the samples has the analytical capacity with adequate limits of detection for each AD.
- Review the lab’s practices regarding reporting of results and fee structure before selecting a lab, to ensure resources are used efficiently and that the data provided will substantively inform the adequacy of controls and housekeeping practices.
